# A 3D printed sheath flow interface for surface enhanced Raman spectroscopy (SERS) detection in flow†

**DOI:** 10.1039/d3an02125d

**Published:** 2024-02-02

**Authors:** Courtney J. Morder, Zachary D. Schultz

**Affiliations:** a Department of Chemistry and Biochemistry, The Ohio State University 140 W. 18th Avenue Columbus OH 43210 USA schultz.133@osu.edu

## Abstract

Surface enhanced Raman spectroscopy (SERS) is an effective technique for detecting molecules in aqueous solutions due to its insensitivity to water, which makes it especially useful for biological samples. Utilizing SERS in flow can aid in a variety of applications such as metabolomics, pharmaceuticals, and diagnostics. The ability to 3D print complex objects enables rapid dissemination of prototypes. A 3D printed flow cell for sheath flow SERS detection has been developed that can incorporate a variety of planar substrates. The 3D printed flow cell incorporates hydrodynamic focusing, a sheath flow, that confines the analyte near the SERS substrate. Since the SERS signal obtained relies on the interaction between analyte molecules and nanostructures, sheath flow increases the detection efficiency and eliminates many issues associated with SERS detection in solution. This device was optimized by analyzing both molecules and particles with and without using sheath flow for SERS detection. Our results show that the flow rates can be optimized to increase the SERS signal obtained from a variety of analytes, and that the signal was increased when using sheath flow. This 3D printed flow cell offers a straightforward method to disseminate this technology and to facilitate online SERS detection.

## Abstract 2

A 3D printed flow cell for SERS detection was developed that incorporated sheath flow to confine analytes near the SERS substrate. The device was optimized by analyzing both molecules and particles with and without sheath flow for SERS detection.

## Graphical abstract

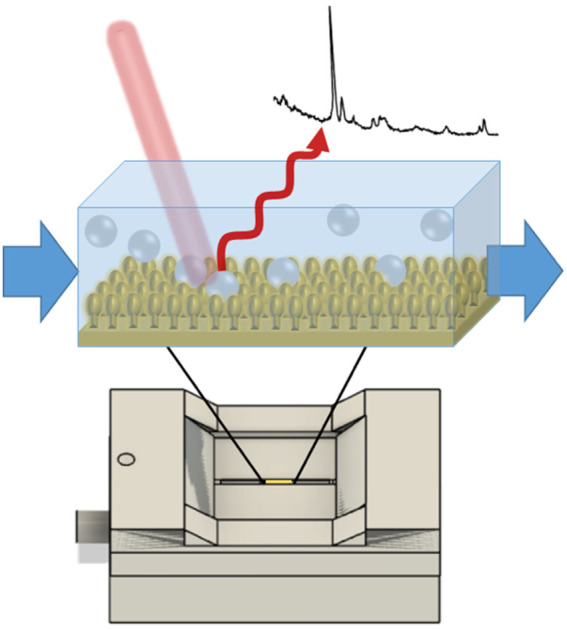

## Introduction

The ability to identify and quantify molecules in flowing solutions is crucial for high throughput analysis in many chemical and biological applications. This opens the door for real time monitoring of samples for diagnostics or process analytical technology. Surface enhanced Raman spectroscopy (SERS) is a sensitive analytical technique capable of detecting low concentrations and even single molecules.^1–4^ Additionally, SERS can provide molecule specific information and is especially useful for analyzing aqueous solutions due to its insensitivity to water. Performing SERS in flowing solutions has been demonstrated for detecting molecules following a chemical separation, such as liquid chromatography or capillary electrophoresis.^5–11^ This allows for real time analysis, minimal sample preparation, and overall faster analysis times for complex mixtures.

SERS incorporates plasmonic, metallic nanostructures to increase the Raman signal observed from analytes. Two common substrates used for SERS analysis include colloidal solutions or planar, roughened surfaces. Performing SERS in flow with colloidal solutions often leads to unpredictable and irreproducible signals, as it is hard to control the formation of aggregates and hotspots.^12–17^ To avoid these issues, roughened planar surfaces offer a more controlled environment. When using these substrates, detection depends on the analyte's ability to diffuse to the surface.^7,18^ In order for the signal enhancement to occur, analyte molecules need to be within a few nanometers of the nanostructured surface.^2,3,19–22^ This can complicate detection of analytes in fluidic channels due to the limited time analytes have to interact with the SERS surface in the detection area. Therefore, minimizing the sample stream to the diffusion layer eliminates the need for an analyte to diffuse through the bulk first. The Schultz lab previously developed a device incorporating hydrodynamic focusing to increase detection efficiency by confining analytes closer to the SERS substrate.^7^

Hydrodynamic focusing utilizes a sheath flow that moves at a higher volumetric flow rate compared to the sample stream.^7,23,24^ The faster moving stream occupies a larger volume in the channel, thus, as the sheath flow rate increases, the sample stream thickness decreases. This minimizes the distance that a molecule needs to travel to reach the SERS surface. The degree of confinement of the sample stream is dependent on several factors, such as the ratio of the sheath and sample flow rates. The Schultz lab has utilized sheath flow for improved SERS detection of neurotransmitters,^25^ peptides,^26^ metabolites in urine,^27^ and sugars.^28,29^

The geometry of the fluidic channels affects the thickness of the confined stream as well, and can be adjusted to improve detection for a selected application.^24,30,31^ When a fluid stream is introduced at an angle relative to the main channel, a component of the velocity is normal to bottom of the channel, which impacts the sample stream. Having a component of the velocity normal to the bottom of the channel means that some of the stream's momentum is going in the vertical direction, which exerts a force onto the sample stream, pushing it towards the bottom of the channel, thus further decreasing the sample thickness.^24,30,31^ However, this can also cause “cusping”, or even splitting, of the sample stream, in which some of the sample stream is pushed to the side walls of the channel. Ligler and coworkers have shown that this “cusping” can be minimized by introducing the sample flow at an angle relative to the main channel, rather than introducing the sheath fluid at an angle.^24,31^ This is because the sheath fluid moves at a faster rate, and will thus impart greater momentum onto the sample stream. It is critical to optimize the flow ratio for the geometry of the microfluidic devices to get the best possible confinement for the desired system. Previous work in the Schultz lab has shown that when introducing the sample fluid and sheath fluid at a 90° angle, a sheath to sample flow ratio of 5 : 1 yielded optimum confinement. At higher flow ratios, the sheath and sample fluids mix, leading to dilution of the sample.^32^ When the sample and sheath fluid are introduced to the channel in parallel, a higher sheath to sample (36 : 1) flow ratio is needed for optimum confinement.^7^ Additionally, as mentioned previously, SERS detection in flow is limited by the ability of a molecule to diffuse to the surface. Thus, these parameters may need to be optimized when detecting analytes with varying properties.

The rise of 3D printing allows for easy dissemination of designs and devices among many researchers. This provides more access to these experiments and facilitates advancements in the field. One of the most attractive 3D printing techniques for microfluidics is stereolithography (SLA) due to its high resolution, high accuracy, and ability to produce objects with low surface roughness.^33,34^ SLA utilizes a vat, photo curable resin, a light source (typically a UV laser), and a movable platform. The configuration used here involves the platform being submerged in the vat of resin, with a UV laser shining through the bottom to polymerize the resin onto the platform. As each layer is polymerized, the platform moves upward so that a new layer can be added on.^33,35,36^ 3D printing is also a cost effective and rapid method for producing microfluidic devices. Previous reports show that these microfluidic devices can be useful for a variety of applications including environmental,^34,36^ biological,^34,37–39^ and electrochemical studies.^36,40–42^ Recently, 3D printing has even been used to produce SERS active substrates.^43,44^ Here we report the development of a 3D printed device for in flow SERS detection that incorporates a sheath fluid to improve detection. This device only costs 2.60 USD to produce, and can easily be modified to accommodate a variety of SERS substrates. The flow parameters were optimized for both molecule and particle detection.

## Experimental

### Materials

Gold and silver substrates were purchased from Silmeco (SERStrate). Ultrapure water (18.2 MΩ cm) was obtained from a Barnstead Genpure system. 4-Mercaptobenzoic acid (MBA), cysteamine, and riboflavin were purchased from Sigma-Aldrich. Phosphate buffered saline (PBS) 1× was purchased from Gibco. Orange tough resin was purchased from Prusa. Isopropanol (IPA), sodium hydroxide, hydrochloric acid, nitric acid, and PTFE tubing (i.d. = 1/32 in) were purchased from Fisher scientific. Carboxylated polystyrene beads (100 nm) were purchased from Interfacial Dynamics Corp. Silver shot was purchased from Kurt J. Lesker. Anodized aluminum oxide filters (AAO) (0.2 μm pores) were purchased from Cytiva. Loctite EA 9017 and Gorilla glue was purchased from McMaster-Carr. Coverslips were purchased from VWR. Clear nail polish was purchased from LA Colors. PTFE thread seal tape was purchased from Lowes.

### 3D printed flow cell preparation

A 3D printed flow cell was designed using Autodesk Fusion 360. The CAD files were then sliced in PrusaSlicer software. These files are provided in the ESI† as .OBJ for use with any CAD software, and as .3MF and .f3d for use with Autodesk Fusion and PrusaSlicer. The flow cell was printed using a Prusa SL1 3D printer with a layer height of 0.035 mm and exposure time of 10 s. The initial layer height was 0.035 mm and initial exposure time was 45 s. The flow cell was printed at an angle to the platform to promote drainage of excess resin and supports were manually added to hold it securely. Immediately after printing, the pieces were moved to a Prusa CW1 for washing and curing. The pieces were washed in IPA for 10 minutes. Then the pieces were removed from the IPA bath and placed back into the CW1 to dry and cure for 3 minutes each.

A glass coverslip (18 mm × 18 mm) was glued to the top piece of the flow cell to seal the channel and provide a viewing window for SERS measurements using gorilla glue. The glue was allowed to cure for 24 hours before use. To attach tubing to the flow cell, 200 μL pipette tips were secured in the sheath flow inlet and outlet, and PTFE tubing was secured to the sample inlet, using orange tough resin and a 405 nm laser pointer. Each channel was filled and rinsed with isopropanol to remove any uncured resin during this process. Each piece was then placed back into the CW1 to cure these connections for 10 minutes. PTFE tubing was then glued into the pipette tips using epoxy. This was allowed to cure for at least 24 hours prior to use. PEEK connections were used to attach syringes to the tubing.

Before and after each use, the flow cell and tubing were cleaned using 1 M nitric acid. 3 mL of 1 M nitric acid was flowed through each channel followed by 100 mL of ultrapure water to remove any residual solutions and contaminants. The threads of the flow cell were wrapped in PTFE thread seal tape before each use to seal the device. Care was taken to prevent the thread seal tape from being on top of the base to ensure the channel wasn't blocked.

### Commercial substrate preparation

Au and Ag substrates from Silmeco were heated on a hotplate at 175 °C for 10 minutes before use, as recommended by the manufacturer.^45^ The substrates were checked for contamination prior to use by collecting SERS spectra and looking for unintended peaks. If needed the substrates were rinsed with 0.1 M hydrochloric acid until the contaminant peaks were not seen, as shown in Fig. S1.† For polystyrene bead experiments, the substrates were soaked in a 10 mM ethanolic solution of cysteamine overnight following heating.

### Thermally evaporated substrate preparation and MBA functionalization

Thermally evaporated silver SERS substrates were prepared using a previously reported protocol.^46^ Briefly, silver shot was evaporated onto anodized aluminum oxide filters with 0.2 μm pores to a thickness of 500 nm. The substrates were soaked in a 5 mM ethanolic solution of 4-mercaptobenzoic acid overnight. The filter was then dissolved by soaking in 0.1 M NaOH for 4 hours. The substrates were then transferred to the base of the 3D printed flow cell and affixed with nail polish on the sides of the substrate around the main channel.

### PBS preparation

1X PBS was pH adjusted using 0.1 M hydrochloric acid or 0.1 M sodium hydroxide. A pH probe (Orion PerpHecT ROSS Combination pH microelectrode, Thermo Scientific) was calibrated at pH 2, 4, 7, and 11 used to monitor the solutions.

### Raman measurements

For experiments using Ag substrates, a homebuilt Raman setup was used equipped with a 632.8 nm HeNe laser (Thor Labs).^11^ A laser power of 0.25 mW with an exposure time of 250 ms was used. 100 spectra were collected in series per sample. 3 series were collected per sample. A 40×/0.8 NA water immersion objective from Olympus was used. The Raman scattering was collected through the same objective and directed to an Andor Shamrock 303i spectrograph with an Andor iDus 401 CCD.

For experiments using Au substrates, a homebuilt Raman setup was used equipped with a 785 nm laser (Oxxius).^37^ The laser was focused onto the samples through a 40x water immersion objective (NA = 0.8, Olympus). The Raman scattering was collected through the same objective and directed to an Isoplane SCT-320 spectrograph with a ProEM 1600^2^ eXcelon 3 CCD detector (Princeton Instruments). Acquisition times of 250 ms were used with a laser power of 0.25 mW. 100 spectra were collected per series, with 3 series collected per sample, and averaged for analysis.

Syringe pumps (Model NE-1000, New Era Pump Systems Inc.) were used to pump all solutions through the flow cell.

## Data analysis

All data was processed in MATLAB R2018B (Mathworks). A peakfitting algorithm was used to fit peaks of interest to a Gaussian lineshape.^47^

## Results

**Fig. 1:**
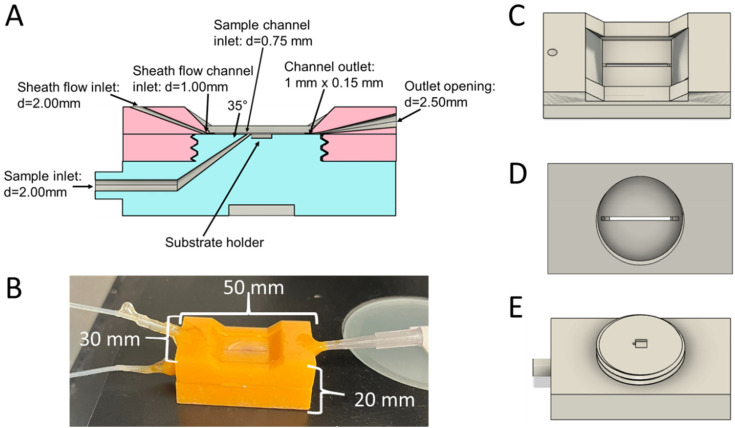
CAD images of the flow cell design. (A) Cross section of the flow cell put together. Top piece is pink and bottom piece is blue. The dimensions of the flow channels are labeled on the figure and show the sample channel entering the main detection channel at a 35 degree angle right before the substrate. (B) Photograph of the flow cell put together and ready to use. (C) CAD image of the top piece showing the sheath flow inlet and main channel through the imaging window. A square coverslip is glued into the center of this piece to seal the channel. (D) CAD image of the bottom side of the top piece. This shows the channel that is sealed by the bottom piece. (E) CAD image of the bottom piece which contains the sample inlet and space for the SERS substrate.

A 3D printed sheath flow cell for SERS detection was developed that can be used to incorporate a variety of SERS substrates, such as commercially available and in house prepared substrates. Fig. 1 shows the CAD diagrams of the flow cell for incorporating commercially available substrates from Silmeco. Fig. S2† shows CAD diagrams for the flow cell when incorporating thermally evaporated substrates prepared in house. The flow cell consists of 2 pieces, a top piece (Fig. 1C and D) and a base piece (Fig. 1E). The top piece contains the sheath flow inlet, main channel, and the outlet. A slot for an 18 × 18 mm coverslip is included in the design to seal the channel, by gluing this on with gorilla glue, and to provide a window for SERS detection. This coverslip needs to be affixed post-printing and curing. The base piece has a slot for the substrate, which can easily be modified to fit any SERS substrate in the CAD design, and the sample inlet. The two pieces have threads for easy assembly. In order to seal the flow cell, the threads need to be wrapped in PTFE tape, ensuring that the tape is only on the threaded part so that the channel does not become blocked. The channel size is 1 mm × 0.20 mm × 21 mm (w × h × l), and only requires 4.2 μL of sample to fill the channel. The outlet for the device is larger than the inlets to alleviate pressure as fluids flow through the cell. The sample inlet is also smaller than the sheath flow inlet to help with confinement to the SERS substrate at the bottom of the channel, so that the sheath fluid can interact on top and on the sides of the sample flow. The dimensions of the device when put together are 30 mm × 20 mm × 50 mm (w × h × l), and the final assembled device is shown in Fig. 1B.

The printed device incorporates a 35° angle between the sample and sheath fluids to improve the confinement by the sheath flow without splitting the sample flow reported with larger confluence angles.^24,30,31^ The sample stream is incident at an angle relative to the main channel, as previous reports show that this minimizes splitting of the sample stream, and the higher momentum of the sheath fluid moving in line with the channel can help carry the sample fluid through the channel as well.^31^ It is critical for the analytes to interact with the surface at the point of SERS detection. A faster moving sheath fluid will occupy more of the channel than the slower moving sample fluid, thus confining the analytes closer to the surface. Previous reports show that having the 2 fluids interact at angles approaching 90° can cause some cusping of the sample fluid or even splitting.^24,31^ This would push more sample molecules towards the walls of the channel and not to the SERS surface. These effects are also more pronounced when faster flow rates are used, or when the sheath fluid is introduced at an angle to the main channel.^24,31,32^ However, previous reports show that having shallow confluence angles between the 2 fluids can further improve the confinement of the sample fluid, with minimal cusping.^24,30,31^

**Fig. 2:**
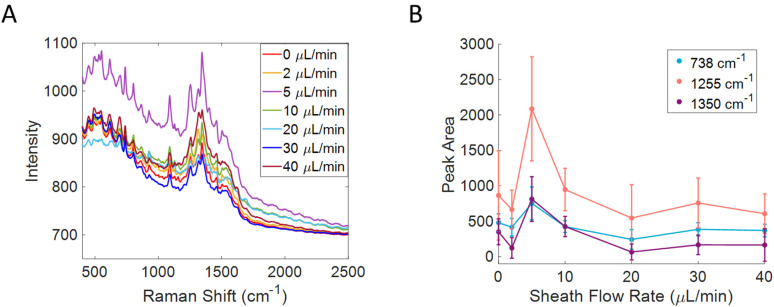
Optimizing flow parameters for molecule detection in 3D printed flow cell using riboflavin as a model analyte. Raw spectra at varying sheath flow rates is shown in (A), while the sample flow rate was kept constant at 1 μL min^−1^. 3 peaks arising from riboflavin were fit and the corresponding peak area was plotted against sheath flow rate (B). The signal was most intense when a sheath flow rate of 5 μL min^−1^ was used.

To determine the effect of the flow ratio on the confinement of small molecules, a 50 μM solution of riboflavin was flowed through the flow cell equipped with a silver substrate from Silmeco. The sheath flow rate was varied while the sample flow rate was kept at 1 μL min^−1^, shown in Fig. 2A. The peak areas of the 738 (C–C bending of benzene ring), 1255 (C=O bending, pyrimidine stretch), and 1350 (C–N–C stretching of pyrazine ring) cm^−1^ bands were tracked and plotted against sheath flow rate.^11,48,49^ By monitoring the peak area as a function of sheath flow rate, there is a clear trend in which the signal was at a maximum when the sheath flow rate is 5 μL min^−1^, as shown in Fig. 2B. When using higher sheath flow rates, the signal starts to decrease, likely due to increased turbulence and washing away of the analyte molecules in the detection region. This could also be due to the shorter dwell time that analytes have to interact with the surface when faster flow rates are used. When the sheath flow is included, the signal increases 2.5 times compared to that without sheath flow. The noise at the optimum flow rate also increases, likely due to increased variability in the number of detected molecules, increased shot noise, and additional signals from heterogeneous enhancements on the substrate. Prior literature indicates signal variation in SERS can be addressed using internal standards and multivariate analysis.^11,50–52^ The optimum flow ratio observed is consistent with previous reports in which the sample stream is introduced at an angle relative to the sheath flow and channel, in which lower flow ratios show the optimum confinement.^30–32^

**Fig. 3:**
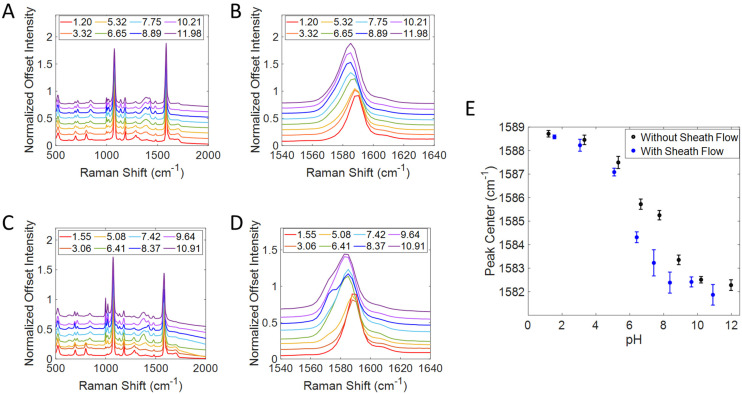
SERS spectra of MBA while flowing PBS solutions over the surface at varying pH values with sheath flow off (A) with a zoom in on the 1585 cm^−1^ band (B), and with sheath flow on (C & D). The center of the 1585 cm^−1^ band was used to create a titration curve (E). The inflection point on this curve is much sharper when the sheath flow was on.

To further analyze the effect of the sheath flow on SERS detection in this device, titration curves were created by taking advantage of the pH response from MBA, shown in Fig. 3. A thermally evaporated Ag SERS substrate was functionalized with MBA before being placed into the 3D printed flow cell. Solutions of PBS at varying pH values were flowed over the surface with the sheath flow rate of 5 μL min^−1^, and without the sheath flow. All spectra were normalized to the 1080 cm^−1^ peak (*ν*_12_ ring breathing mode) and offset for clarity in Fig. 3A and C.^53–55^ The position of the *v*_8a_ ring breathing mode at 1585 cm^−1^ was monitored throughout the runs, by using a peakfitting algorithm in Matlab,^47^ as this peak is known to red shift with increasing pH.^54,56–58^ By monitoring this peak frequency, the confinement of the analyte solution can be assessed through the local pH without concern about analyte finding hotspots on the surface. A zoom in on this region for the runs without a sheath flow and with a sheath flow are shown in Fig. 3B and D, respectively. The average peak center and standard deviations of the 1585 cm^−1^ band (*v*_8a_ ring breathing mode) were then plotted against pH in Fig. 3E. The titration curve with sheath flow on shows a much sharper inflection than the one with sheath flow turned off. This difference suggests that we are more effectively controlling the pH at the substrate surface when incorporating sheath flow. Without sheath flow, we still observe the shift in the 1585 cm^−1^ band as the pH of the sample flow changes, however this occurs more gradually than when sheath flow is used. A more pronounced shift in this band near a pH of 5.5 indicates that more MBA molecules in the detection region are experiencing the change in pH than those on the surface when sheath flow is not used. The shoulder observed in the basic pH runs in Fig. 3D is likely due to some decarboxylation of the MBA, as characteristic peaks of thiophenol grow in at 1003, 1026, and 1576 cm^−1^. It is known that MBA molecules can be decarboxylated more easily in high pH environments.^59–61^

**Fig. 4:**
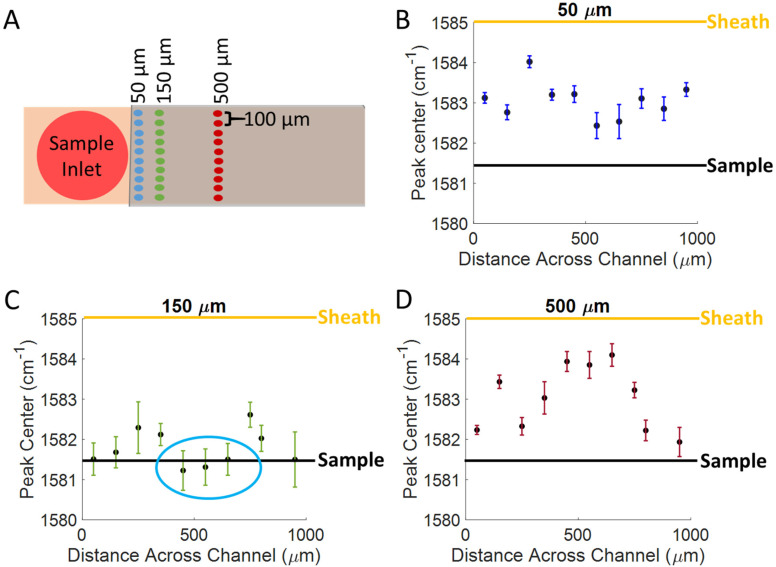
Distance dependence of sheath flow focusing effects. SERS spectra were acquired on an MBA functionalized substrate while a sheath fluid of pH 5.63 and a sample fluid of pH 11.55 were flowed over the surface. (A) Diagram indicating where spectra were collected in relation to the sample inlet. Spectra were acquired every 100 μm across the channel at (A) 50 μm, (B) 150 μm, (C) 500 μm from the inlet. The black line represents the expected peak center when detecting at the sample fluid pH and the yellow line represents the expected peak center when detecting at the sheath fluid pH.

To better understand the confinement of the analyte within the channel, spectra were acquired on MBA functionalized SERS substrates by using water as a sheath fluid, with a pH of 5.63, and a sample fluid of PBS at pH 11.55. The flow rates were 1 μL min^−1^ and 5 μL min^−1^ for the sample and sheath fluids, respectively. Spectra were acquired across the channel in 100 μm increments starting at one of the side walls and moving towards the other. By measuring across the channel, evidence of cusping can be monitored. Spectra were acquired across the channel and at various distances from the sample inlet, and are shown in the diagram of Fig. 4A. By monitoring the position of the 1585 cm^−1^ peak, we are able to determine whether the analyte is confined in the sample solution (pH = 11.55), the sheath fluid (pH = 5.63), or in a mixed intermediate pH region, as the position of the 1585 cm^−1^ peak should correspond to the pH experienced at each location. We expect the peak center to be between 1581 and 1582 cm^−1^ where the sample fluid is confined over the substrate based on the titration curve in Fig. 3E, due to the sample fluid having a basic pH (11.55). Since the sheath fluid has a pH of 5.63, we expect for the peak center to be between 1585 and 1586 cm^−1^ in places where the sheath fluid is interacting with the surface the most. The expected peak positions for the sheath and sample fluids are indicated by the yellow and black lines, respectively, on the plots in Fig. 4B–D. The results indicate that the sample is confined to the center of the channel, approximately 400–600 μm from either edge, and 150 μm from where the sheath and sample fluids interact, shown by the blue circle Fig. 4C. The results in Fig. 4 show that there is an optimal location in the flow cell for the best confinement, and there is likely mixing or inconsistent transport of analyte molecules to the surface in other areas. This optimal location is consistent with what many others have reported when using sheath fluids, as the best confinement is usually within 200 μm from the point of interaction between the 2 fluids.^7,24,30–32^ At 50 μm from the inlet, the peak center in the middle of the channel is between 1582 and 1583 cm^−1^ which suggests that some analyte molecules are reaching the surface, but there is still some incomplete transport. At 150 μm from the inlet most of the signal is close to the expected peak center value (1581–1582 cm^−1^), with the closest points being in the center of the channel (400–600 μm), suggesting that this is where the highest efficiency of transporting molecules to the surface occurs. After 500 μm of travelling through the flow cell, the peak center is closer to 1585 cm^−1^, suggesting that very little analyte is reaching the surface, and that the sheath fluid is interacting with the surface more. This difference is likely because the sheath fluid is mixing with the sample at this point.

**Fig. 5:**
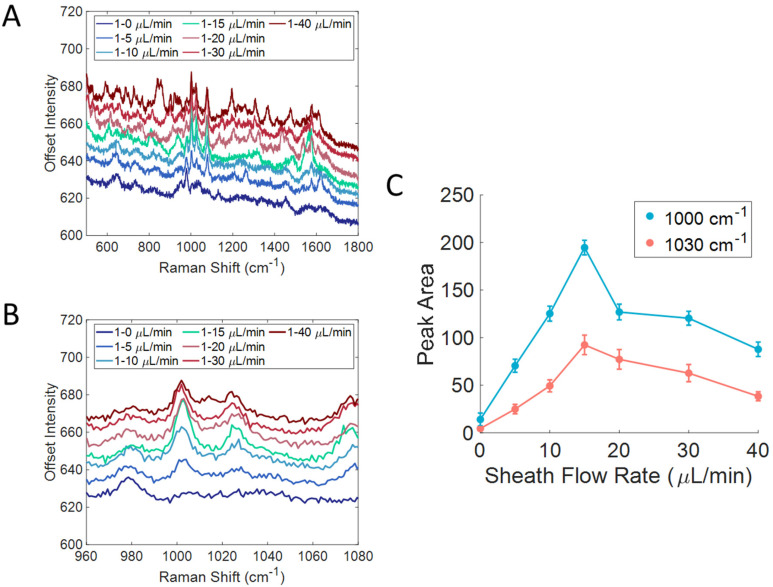
Optimizing flow parameters for particle detection using 100 nm carboxylated polystyrene beads. Raw spectra at varying sheath flow rates is shown in (A), while the sample flow rate was kept constant at 1 μL min^−1^. (B) Zoom in on the key features arising from polystyrene at 1000 and 1030 cm^−1^ corresponding to the ring breathing and ring stretching modes. (C) The peak area of these 2 features was plotted against sheath flow rate to show how the signal improves with increasing sheath flow until it reaches a maximum at 15 μL min^−1^.

We further investigated the flow conditions in this device for the detection of particles. 100 nm carboxylated polystyrene beads (1 wt%) were flowed over an Au substrate from Silmeco that had been functionalized with cysteamine, shown in Fig. 5A. The cysteamine monolayer was adsorbed to promote interactions with the carboxyl group on the particles and displace contaminants on the SERS substrate. The sample flow rate was kept constant at 1 μL min^−1^, but the sheath flow rate was changed to determine the best ratio for particle detection. Fig. 5B shows a zoom in of the 1000 and 1030 cm^−1^ bands that correspond to the ring breathing and ring stretching modes of polystyrene, respectively.^18^ Without sheath flow, signal from the polystyrene beads was not seen, however the signal started to grow in as the sheath flow rate increased. Fig. 5C shows the peak area of those bands plotted against sheath flow rate. This plot shows that the signal was the most intense with a sheath flow of 15 μL min^−1^, and then the signal started to decrease, similar to the trend seen with molecule detection in Fig. 2B. The need for a higher sheath flow rate when detecting particles likely has to do to their size compared to small molecules. A higher flow rate will provide a larger force onto the sample fluid, thus making the sample layer smaller, and leads to a smaller distance that the particles need to diffuse to the surface. A larger force is likely needed since particles are larger in size and have smaller diffusion coefficients. It has been previously reported that interaction with the surface is important for the detection of polystyrene beads.^62^ The larger force needed may arise from the size of the beads inhibiting intercalation within the Silmeco substrate pillars; however, by being close to surface, sufficient enhancement is generated to enable detection. Prior work has shown that it is possible to detect lentiviruses of similar size to the polystyrene beads using Silmeco substrates.^37^

The compatibility between the 3D printed device and various solvents was further studied to determine which types of samples could be analyzed with this device. The experimental details for this experiment are in the ESI.†Table 1 shows a list of the solvents tested and whether or not there were any changes to the solvents or 3D printed objects. The resin was resistant to acids and bases, but was not compatible with organic solvents. The 3D printed objects cracked or fell apart after exposure to all organic solvents tested, including acetone, DMSO, ethanol, and isopropanol. This study suggests that cleaning the channels with 1M nitric acid won't affect the flow cell over time, but should remove any residual analyte that remains. All Raman spectra and photographs of the objects before and after exposure to the solvents are shown in the ESI.† Fig. S3† shows the spectra and photographs from the solvents that did not affect the cured objects. Fig. S4–S6† show the effects of the organic solvents on 3D printed objects.

**Table 1:** Summary of compatibility between cured orange tough resin and various solvents

Solvent	Raman spectral changes	Visible changes
Acetone	New features at: 573 999, 1300 cm^−1^	- Solution turned orange
		- 3D printed object fell apart
1 M ammonium hydroxide	No	No
Bleach, 8.25% hypochlorite	No	No
Dimethyl sulfoxide (DMSO)	Overall intensity decreased	- Solution turned orange
		- 3D printed object fell apart
Ethanol	Overall intensity decreased	3D printed object cracked after drying
Isopropanol	- Intensity decrease	3D printed object cracked after drying
	- New feature at 940 cm^−1^	
1 M hydrochloric acid	No	No
1 M nitric acid	No	No
1 M sodium hydroxide	No	No
1 M sulfuric acid	No	No
Water	No	No

## Discussion

The 3D printed flow device shown here can easily be disseminated between labs and has a straightforward setup to facilitate SERS in flow experiments. Incorporating a sheath flow also improves detection in flow due to the improved transport of analyte molecules to the substrate surface. The sheath flow can be optimized to decrease the sample layer height, which means that the molecules have a smaller distance to travel to reach the SERS substrate. The transport of analytes also appears to be related to their size. For small molecules it was determined that a 1 : 5 sample to sheath flow ratio produced the best signal, and for larger particles (100 nm polystyrene beads) it was found to be 1 : 15. We hypothesize this difference is due to lower diffusion by larger particles. The larger flow ratio is needed for particle detection due to their size, as larger molecules have smaller diffusion coefficients, and thus are slower to diffuse to the surface. Prior work has indicated that adsorption to the surface is a key factor for SERS detection in fluids.^62^ The results here suggest that flow rates also impact this interaction possibly due to differences in diffusion. It is beneficial to use a faster sheath flow to confine the sample stream to a smaller thickness, meaning that the particles need to travel a smaller distance to reach the surface. However, small molecules can diffuse faster, which is why a slower sheath fluid is needed to optimize the confinement, as they can travel a larger distance than particles can within the same amount of time. The higher flow rates may impart additional momentum to particles, which is not experienced by molecular analytes. The signal for both riboflavin and polystyrene beads increased when using a sheath fluid compared to the signal when no sheath fluid was used, and while the riboflavin signal decreased at higher flow rates, the particles were detected more efficiently at the higher sheath flow rates. It will remain important to assess how analyte size impacts detection efficiency, which may provide additional avenues for analyte selectivity in the future.

Introducing the sample flow to the main channel at an angle was reported by Ligler and coworkers to result in better confinement.^31^ They examined how the fluidic profiles changed based on whether the sheath or sample flows were in line with the main channel. They observed a flatter profile and minimal “cusping” when the sample flow was introduced at an angle to the main channel.^24,31^ This is likely due to the sheath flow having a higher momentum, which would be advantageous to keep in line with the main channel because the sample flow would not have enough momentum to penetrate the sheath flow, thus the sheath flow momentum would help carry the sample stream through the channel. When the sheath flow is introduced at an angle relative to the main channel, its large momentum can penetrate the sample stream, or even split it, which would push most of the analyte to the walls of the channel rather than to the SERS substrate. They also report that the angle of interaction between these two streams can impact the shape of the flow profile. At large confluence angles, such as 90° and 180°, the sample stream is pushed closer to the outer walls instead of being confined to the bottom of the channel. When an angle of 45°was used, the sample stream's profile was much flatter and confined to the bottom of the channel.^31^ In Fig. 4, the optimized detection point reported here shows no evidence of cusping. Considering these prior reports, the spatial limitations, and the printing capabilities for producing this 3D printed device, we found an angle of 35° to minimize “cusping” effects while confining the analyte molecules to the surface. With improved printing technology, it may be possible to further optimize the channel configuration, though significantly larger improvements are not expected.

Our results show better transport of analyte molecules and confinement of the sample solution at the surface when the sheath flow is used, compared to when only the sample is being flowed through the device. Having improved transport means that lower sample concentrations can be analyzed in flowing solutions, thus enabling trace detection in flow by SERS. Flow through SERS analysis enables real time monitoring and is an attractive detector for liquid based separations.^5–11^ Incorporating a sheath flow has been shown to wash analyte molecules away from the surface to allow for regeneration of the SERS surface.^7^ This ensures that multiple analytes can be analyzed sequentially while using the same SERS substrate, as long as high concentrations are not used to prevent fouling of the surface.^11,26,63^

The ability to 3D print the interface removes much of the technical expertise required to implement sheath flow SERS. The 3D printed flow device reported here requires one day of initial production, which consists of 7 hours and 15 minutes to print, followed by 16 minutes of post-print processing using the 3D printer reported here. After this process, the tubing and coverslips can be attached and left to dry overnight. For every use after this initial setup, the flow cell can be rinsed with 1 M nitric acid followed by water to clean the channels of any residual analyte, and can then be put together within a few minutes. With proper care and use, this flow cell can be reused indefinitely without the need to print a new one for each experiment. The reported design enables straightforward exchange of substrates, which need to be replaced more often, likely after each experiment. Irreversible adsorption to SERS substrates is a known problem;^64,65^ however, this ability to exchange substrates in the flow may facilitate SERS experiments with reusable substrates^66–69^ or with improved cleaning methods.^70^ Some precautions need to be taken, as the resin used for 3D printing can react with some chemicals. Organic solvents, will react with the resin causing destruction of the flow cell. Table 1 lists solvents that were tested for compatibility with the orange tough resin used for this flow cell. Another consideration is the compatibility between the sample and the resin, as many resins used for SLA printing are toxic and cannot be used with cells or other biological samples.^33^ However, the resin used in this study was shown to be compatible with most solvents tested, and did not affect SERS detection of the analytes used. The development of new 3D printing resins may enable testing in new solvent environments. 3D printing allows for easy and rapid dissemination of prototypes which improves the ease with which this technology can be shared and developed. This could increase the number of people that have access to these types of experiments and lead to more advancements in the field. These designs can also easily be modified to fit a variety of substrates and tubing connections to fit many applications. Lastly, performing SERS in flow allows for online coupling to liquid phase separation techniques suggesting that this device could be used for aqueous based separations. Straightforward SERS detection in flowing solutions can lead to real time analysis in a variety of fields, such as diagnostics, environmental studies, and pharmaceutical development.

## Conclusion

A 3D printed sheath flow cell was developed for improved SERS detection in flowing solutions. This device was optimized for the detection of a variety of analytes, such as small molecules and particles (100 nm). Incorporating a sheath flow improves transport of analyte molecules to the SERS surface and increases the detection efficiency in SERS experiments. The optimum sample: sheath flow ratio was determined to be 1 : 5 μL min^−1^ for small molecules, such as riboflavin, and 1 : 15 μL min^−1^ for 100 nm polystyrene beads. The difference in these ratios is likely due to the difference in diffusion constants or size differences for these analytes. Increasing the sheath flow for larger molecules can impart a larger force pushing them closer to the surface and decreasing the distance needed to reach the SERS surface. The ability to 3D print a device enables faster dissemination of this technology, and increases the accessibility of performing these SERS experiments in flow. Additionally, the design can be easily adjusted to incorporate a variety of substrates if the dimensions are known. We show here that the device was capable of using both in-house prepared and commercially available substrates. Additionally, the ability to perform SERS in flow is useful for coupling this technology to separation techniques, which can impact many applications such as pharmaceuticals, diagnostics, and environmental studies.

## Conflicts of interest

ZDS is an inventor on US Patent 9,804,093, “Ultrasensitive SERS flow detector”, describing the initial sheath-flow SERS detection interface.

## Supplementary Material

AN-149-D3AN02125D-s001

AN-149-D3AN02125D-s002
