# Supplementary material for: A 3D printed sheath flow interface for surface enhanced Raman spectroscopy (SERS) detection in flow
Source: Analyst. 2024 Feb 2;149(6):1849–60. doi: 10.1039/d3an02125d (PMC10926779; doi:10.1039/d3an02125d)
Supplement: AN-149-D3AN02125D-s001 [file AN-149-D3AN02125D-s001.pdf]

Electronic Supplementary Material (ESI) for Analyst.
This journal is © The Royal Society of Chemistry 2024

Supplemental Information: Development of a 3D printed device for online surface enhanced Raman spectroscopy (SERS) detection

Courtney J. Morder and Zachary D. Schultz*

Department of Chemistry and Biochemistry, The Ohio State University, 140 W. 18th Avenue, Columbus, OH 43210, USA

*corresponding author email: schultz.133@osu.edu

Table of Contents

Resin- Solvent compatibility experimental details S-2

Figure S1: HCl cleaning of substrates S-3

Figure S2: CAD images of the flow cell for use with thermally evaporated substrates S-3

Figure S3: Compatibility of resin and solvents without noticeable changes S-4

Figure S4: Compatibility of resin and DMSO and Ethanol S-5

Figure S5: Compatibility of resin and isopropanol S-6

Figure S6: Compatibility of resin and Acetone S-6

Experimental Details

Materials

Orange tough resin for 3D printing was acquired from Prusa. Acetone, Dimethyl sulfoxide (DMSO), and ammonium hydroxide were acquired from Fisher Scientific. Isopropanol and sulfuric acid were acquired from VWR. Hydrochloric acid, nitric acid, and sodium hydroxide were purchased from Sigma Aldrich. Ethanol was purchased from Decon Laboratories. Bleach was obtained from Clorox. Ultrapure water (18.2 MΩ) was acquired from a Barnstead Genpure system.

Methods

3D printed calibration objects were printed from Prusa's calibration files (Prusa Clay Army). The objects were washed and dried in a Prusa CW1 curing and washing machine. The objects were washed in isopropanol for 10 mins, dried for 3 mins, and then cured for 3 mins. The final objects were placed into various solvents to soak overnight. The solvents included in this study were: acetone, DMSO, ethanol, isopropanol, bleach, ultrapure water, 1M hydrochloric acid, 1M sulfuric acid, 1M nitric acid, 1M sodium hydroxide, and 1M ammonium hydroxide.

Raman spectra were collected using a Snowy Range IM-52 instrument with 638 nm excitation. A calibration object was placed into a vial and then 2 mL of a solvent was added, following the curing step. This was done for each of the solvents studied, and the vials were left for 24 hours. After 24 hours, 1 mL of the solvent was removed and Raman spectra were collected. 3 spectra per sample was collected with an acquisition time of 2 s and laser power of 31 mW. The acquisition time of acetone, DMSO and isopropanol was 0.5 s to avoid saturating the detector. Raman spectra of the pure solvents were also collected using these parameters to use for comparison. The vials and 3D printed objects were also visually inspected for any changes. The 3D printed objects were removed from the solvents and allowed to dry for 3 hours, and were then visually inspected for changes.

Data Analysis

All Raman spectra were processed in MATLAB R2018B (Mathworks). Spectra were baseline corrected using a rolling circle filter with a radius of 100,000.[1]

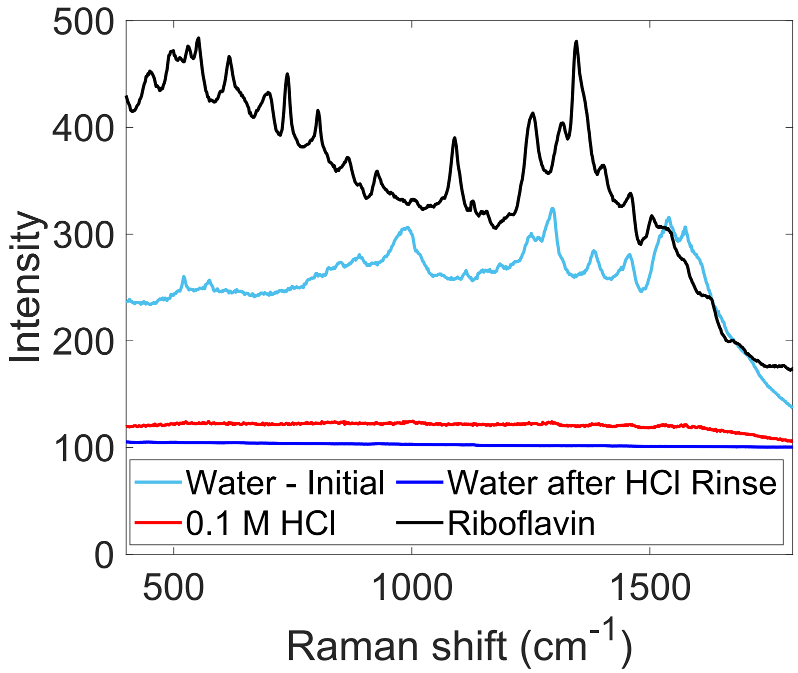

Figure S1. Raman spectra of Ag Silmeco substrate during HCl cleaning. Initial spectra of the substrates are collected with only water on the surface (light blue trace) and some contaminant peaks are observed. To remove the contaminants, 0.1 M HCl is flowed over the surface until the peaks are no longer observed (red trace). The substrates are then flushed with water again (dark blue trace) and then the analyte can be flowed over the surface (black trace).

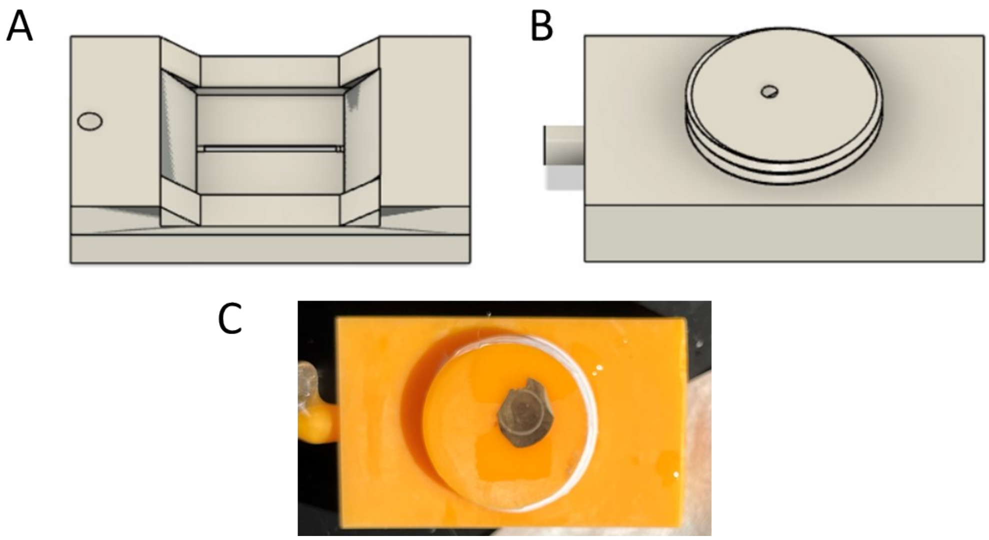

Figure S2. CAD images of the flow device for incorporating thermally evaporated substrates prepared in house. The top piece (A) is the same for both types of substrates, but the slot for the Silmeco substrates is removed so that the thermally evaporated substrates can be affixed at the inlet on the base (B) A photograph of the base with a silver thermally evaporated substrate is shown in (C).

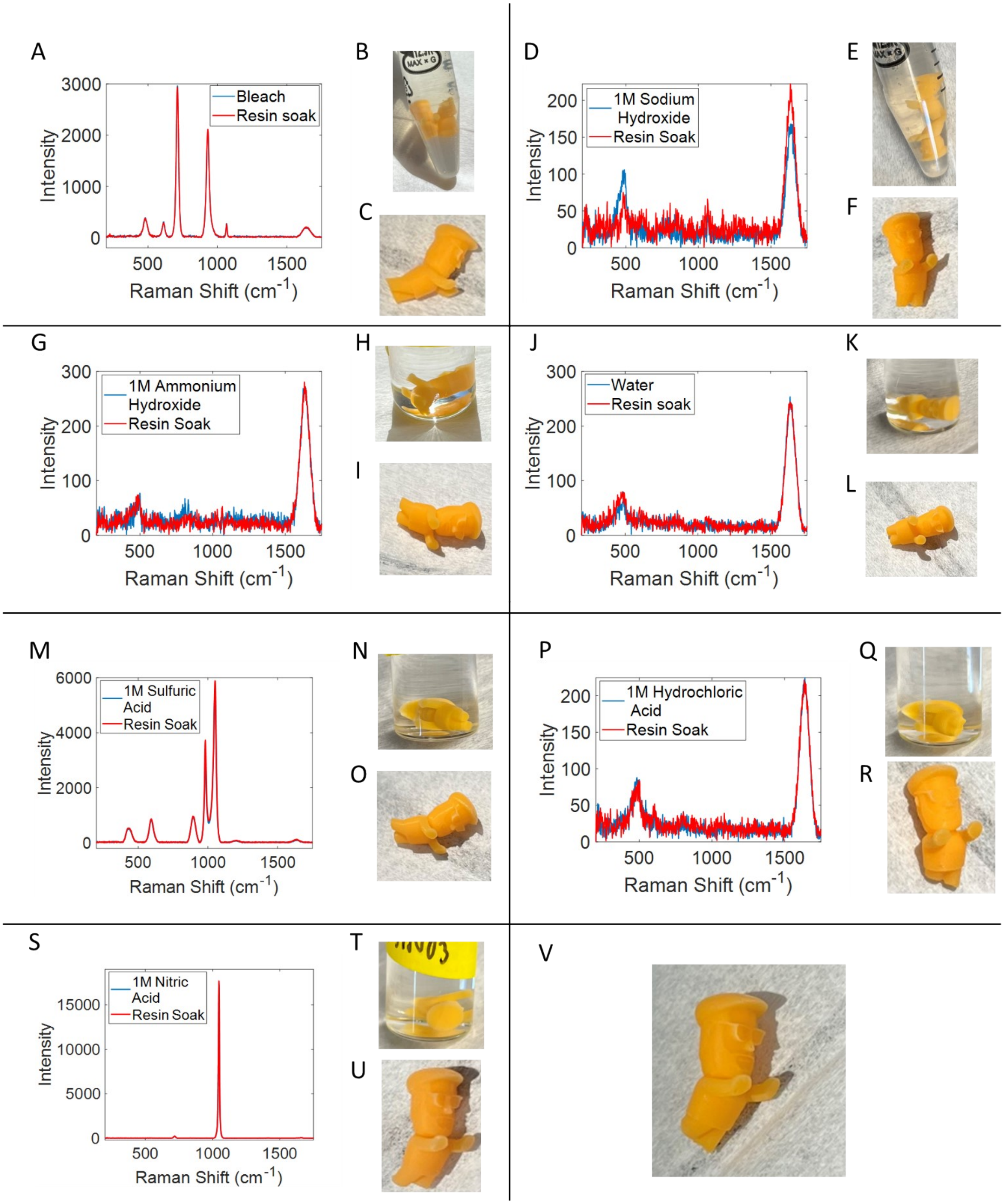

Figure S3. Testing compatibility between cured 3D printing resin and various solvents. The solvents shown here did not show changes in Raman spectra or visually after cured objects were soaked for 24 hours. Solvents tested include Bleach (A,B,C), 1 M sodium hydroxide (D,E,F), 1M ammonium hydroxide (G,H,I), water (J,K,L), 1M sulfuric acid (M,N,O), 1M hydrochloric acid (P,Q,R), and 1 M nitric acid (S,T,U). The photograph in V is of the object without soaking in a solvent as a reference. The Raman spectrum of each pure solvent is shown in blue and the red trace is of the solvent after a 3D printed object was placed in it for 24 hours. The top photograph for each solvent is of the solution after 24 hours and the bottom photograph is of the object after it was removed from the solvent and dried.

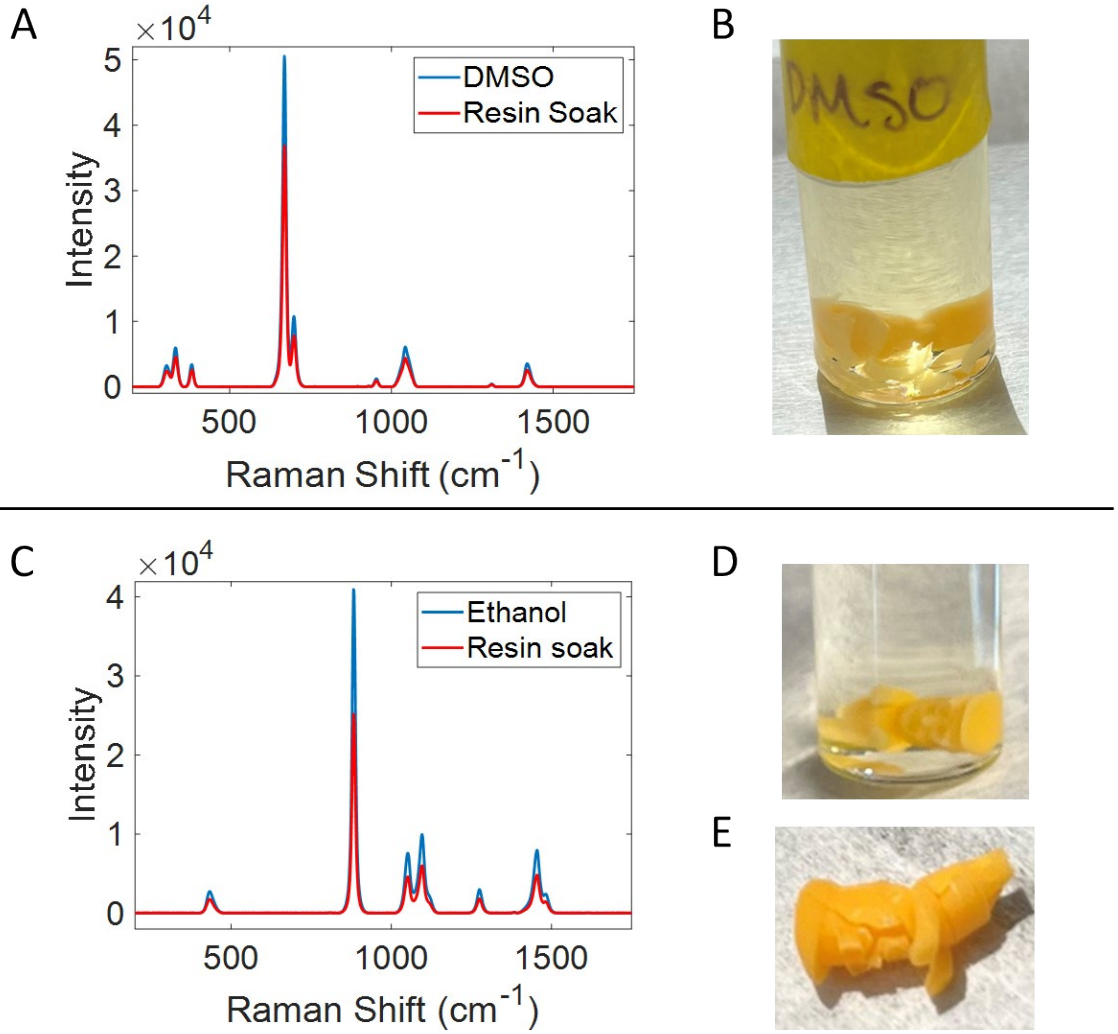

Figure S4. Compatibility between cured 3D printing resin and dimethyl sulfoxide (A&B) and ethanol (C,D,E). Raman spectra of the pure solvents are in blue and the spectrum of the solvent after an object was placed in it for 24 hours is in red. Spectra after soaking a 3D printed object for 24 hours showed a decrease in overall intensity. The object began to fall apart after soaking in DMSO for 24 hours and the solution changed to a faint orange color as shown in photograph B. Photograph D shows the object still intact while in solution, but after the object was removed and dried, it began to crack.

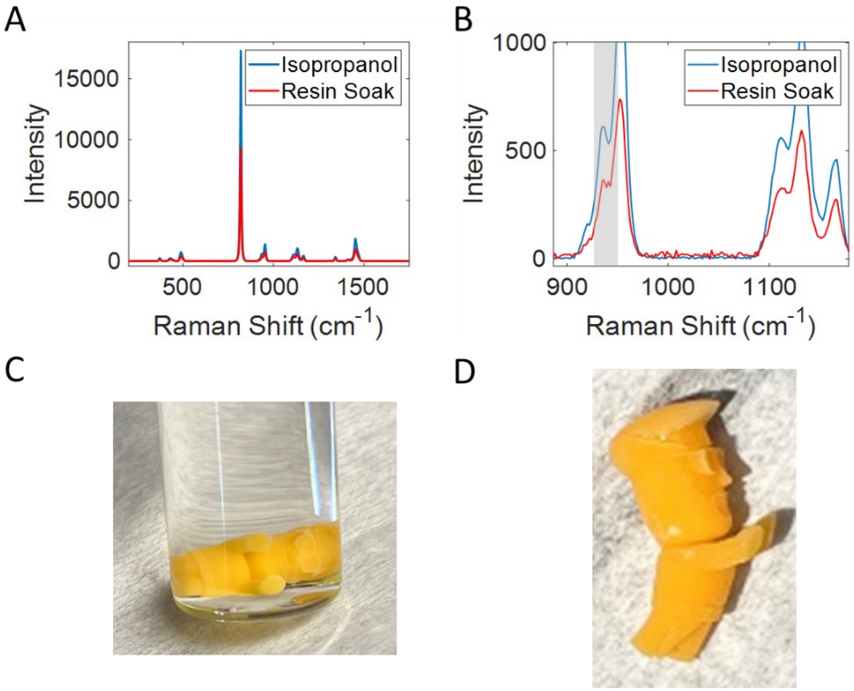

Figure S5. Raman spectra (A) of isopropanol after a 3D printed object was placed into the solvent for 24 hours. An overall decrease in intensity was seen and a small peak around 940 cm^{-1} is observed (B). No visual changes were observed while the object was in solution (C), but after it was removed and dried it began to crack (D).

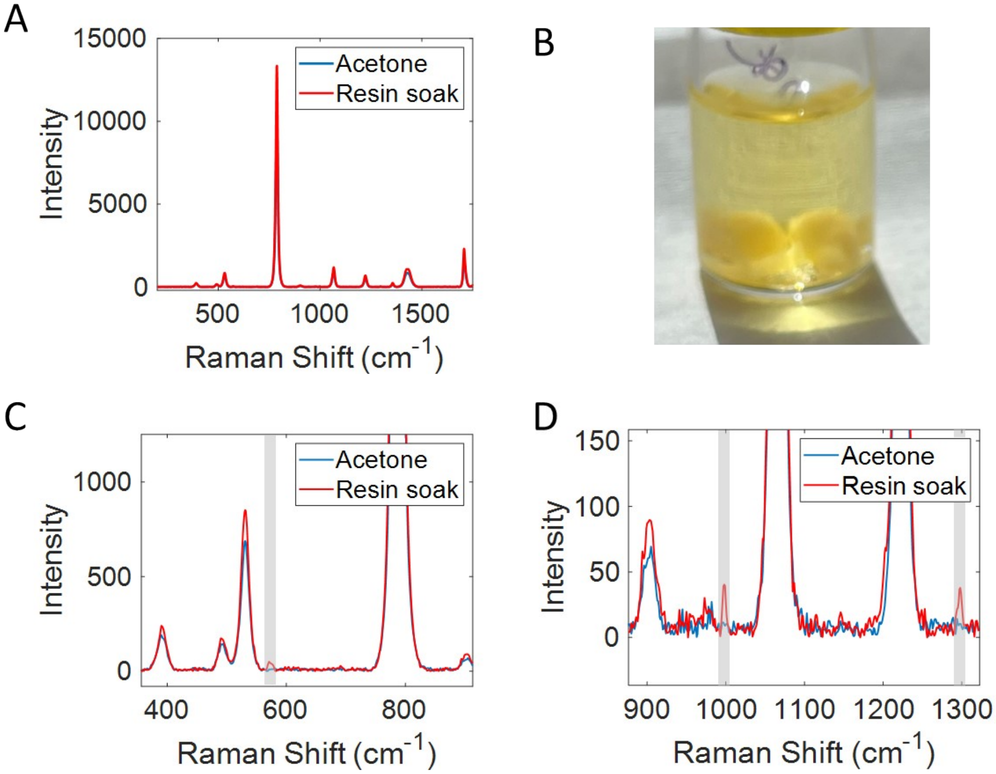

Figure S6. Compatibility between 3D printed objects and acetone were analyzed with Raman spectroscopy (A) and visually (B). The object began to lose color, which was transferred to the solution. The object also became sticky and fell apart when touched. Some new spectral features were observed in the Raman spectra of the acetone solution (C&D). These features include peaks at 573, 999, and 1300 cm^{-1}.

References

(1) Brandt, N. N.; Brovko, O. O.; Chikishev, A. Y.; Paraschuk, O. D. Optimization of the Rolling-Circle Filter for Raman Background Subtraction. *Applied Spectroscopy* **2006**, *60* (3), 288-293. DOI: 10.1366/000370206776342553.
